# Research on Microstructure and Mechanical Properties of Rheological Die Forging Parts of Al-6.54Zn-2.40Cu-2.35Mg-0.10Zr(-Sc) Alloy

**DOI:** 10.3390/ma13245591

**Published:** 2020-12-08

**Authors:** Hansen Zheng, Zhifeng Zhang, Yuelong Bai, Yongtao Xu

**Affiliations:** 1 National Engineering & Technology Research Center for Non-ferrous Metal Matrix Composites, GRINM Group Co., Ltd., No.11, Xingke East Street, Yanqi Economic Development Zone, Huairou District, Beijing 101407, China; zhs200559@163.com (H.Z.); bai_yuelong@163.com (Y.B.); xyt93upcgrinmustb@163.com (Y.X.); 2 GRINM Metal Composites Technology Co., Ltd., No.11, Xingke East Street, Yanqi Economic Development Zone, Huairou District, Beijing 101407, China; 3 General Research Institute for Nonferrous Metals, No.2, Xinjiekouwai Street, Beijing 100088, China

**Keywords:** high-strength aluminum alloy, internal cooling with annular electromagnetic stirring melt-treatment, rheological die forging, mechanical properties

## Abstract

High-strength aluminum alloy (mainly refers to the 7xxx series) is the optimum material for lightweight military equipment. However, this type of aluminum alloy is a wrought aluminum alloy. If it is directly formed by traditional casting methods, there will inevitably be problems such as coarseness, unevenness, looseness, and hot cracking in the structure, which will greatly affect the final performance of the part. Based on the internal cooling with annular electromagnetic stirring (IC-AEMS) method, a new technology of rheological die forging is developed in this paper, and the scale-reduced parts of a brake hub of Al-6.54Zn-2.40Cu-2.35Mg-0.10Zr aluminum alloy were prepared. The influence of IC-AEMS and the addition of rare element Sc on the structure and mechanical properties of the parts was studied. An optical microscope and scanning electron microscope (SEM) were used to observe the microstructure evolution, energy dispersive spectroscopy (EDS) was used to analyze the phase distribution and composition, and the mechanical properties of the parts were tested by uniaxial tensile tests. The results show that the addition of Sc element can effectively refine the grains and improve the strength and elongation of the material; the application of IC-AEMS improves the cooling rate of the melt, increases the effective nucleation rate, and the grains are further refined. Through process optimization, scale-reduced parts of a brake hub with good formability and mechanical properties can be obtained, the ultimate tensile strength is 597.2 ± 3.1 MPa, the yield strength is 517.8 ± 4.3 MPa, and the elongation is 13.7 ± 1.3%.

## 1. Introduction

High-strength aluminum alloy (7xxx series deformed aluminum alloy) has the characteristics of low density, high strength, and good processing performance. It is widely used in aerospace and civil industries. It is one of the main structural materials in the aerospace and transportation industries [1]. In recent decades, scholars around the world have conducted a lot of research on the composition, processing technology, the heat treatment process, and their effects on the performance of high-strength aluminum alloys [2], and have made certain progress, greatly promoting the application of this type of material. High-strength aluminum alloys are mainly based on Al-Zn-Mg-Cu alloys. Al-Zn-Mg-Cu alloys were developed based on the research of Al-Zn-Mg alloys by German scientists W. Sander and K.L. Meissner from 1923 to 1926 [3]. They have high strength and plasticity, and have high fracture toughness and stress corrosion resistance, but due to their high degree of alloying, they are prone to segregation during the casting process, the tendency towards hot cracking is serious, and the casting fluidity is poor. Usually, the casting billet is subsequently subjected to plastic deformation processing to eliminate casting defects and refine the grains, and then can be put into use after heat treatment for strengthening [4,5,6]. It is difficult to form with traditional casting methods such as die casting or gravity casting. Liquid die forging (also often called squeeze casting) technology combines the characteristics of casting technology and forging processing. It is a green molding technology with a shorter process, higher efficiency and lower cost. It is characterized by a wide range of material selection, small forming deformation force, low processing energy consumption, uniform and dense forging structure, and good mechanical properties [7,8,9,10]. The method is to pour a quantitative amount of molten metal into the concave die cavity, close the die when the metal is about to solidify, and the melt is solidified under pressure to obtain the desired shape forging. Compared with traditional die-casting, liquid die forging parts are more thoroughly fed, and it is easier to eliminate various defects; compared with hot die forging, it is easier to form, has less forming force, and can produce more complex shapes. Therefore, liquid die forging technology has attracted widespread attention [11,12,13,14,15].

The use of liquid die forging technology to directly achieve near-net shape forming of the 7xxx series wrought aluminum alloy can greatly improve production efficiency and save costs. Many scholars have studied the influence of liquid die forging process parameters on the mechanical properties of 7xxx aluminum alloy forming parts [16,17,18,19]. It is generally believed that the mechanical properties of liquid die forging parts increase with the increase in pressure; usually, forming pressure is 50–100 MPa, and the casting temperature is between 650 and 730 °C, and the die temperature is between 150 and 250 °C. Before casting, stirring the melt is a common physical method for grain refinement. Compared with mechanical stirring, electromagnetic stirring (EMS) can make the metal melt flow spontaneously, reduce the impurities and gases that may be introduced by mechanical stirring, and obtain a purer melt while also making the temperature field and composition field of the melt more uniform. Wang [20] studied the structure and properties of 7075 high-strength aluminum alloy squeeze castings. Adding Sc and Zr elements to the melt can effectively refine the grains, but the hot cracking is serious and the bimodal structure is prone to appear. He [21] developed the multi-annular electromagnetic stirring (M-AEMS) method, which realized the processing of a large-volume 7075 high-strength aluminum alloy melt, adding Sc and Zr elements to the melt, and using liquid die forging technology to realize the near-final forming of the track shoe. However, there is a phenomenon of uneven organization; Guan [22] proposed the internal cooling with annular electromagnetic stirring (IC-AEMS) method, which not only reduces the influence of the skin effect on melt stirring, but also improves the shear strength. At the same time, the heat transfer effect of the cooling mandrel makes the heat dissipation of the melt in the crucible more uniform and rapid. It is more conducive to the nucleation and refinement of grains, but there is no research to achieve near-net shape forming of high-strength aluminum alloys by this method.

In general, there are still few studies on the direct casting into parts of highly alloyed Al-Zn-Mg-Cu aluminum alloys. In order to save costs and shorten the process, and to obtain near-net shape forming parts with performance equivalent to forgings, this paper uses liquid die forging technology combined with electromagnetic stirring melt processing, and proposes a rheological die forging method. The effects of different conditions on the structure and properties of high alloyed Al-Zn-Mg-Cu aluminum alloy casting parts were investigated. An optical microscope (OM) and scanning electron microscope (SEM) were used to observe the microstructure evolution, and the phase distribution and element composition were analyzed by energy dispersive spectroscopy (EDS). In addition, the mechanical properties of the parts were studied through static tensile experiments.

## 2. Materials and Methods

### 2.1. Materials

Because Fe and Si elements are prone to forming Al_7_Cu_2_Fe and Mg_2_Si coarse and hard phases in Al-Zn-Mg-Cu alloys, and they cannot be solid-dissolved into the matrix in the subsequent heat treatment, which will reduce the mechanical properties of the alloy. Therefore, Fe and Si are harmful elements and should be reduced as much as possible. Meanwhile, Mn, Ti, and Cr elements are unnecessary alloying elements for 7xxx aluminum alloy, so high-purity Al, Mg, Zn, and Al-50Cu, Al-5Zr, Al-2Sc master alloy are selected to configure 6.54Zn-2.40Cu-2.35Mg-0.10Zr(-Sc) alloy. The chemical composition of the alloy was measured by a floor-standing electric spark direct reading spectrometer (FOUNDARY MASTER PRO, Oxford Instruments, Abingdon, UK), as shown in Table 1.

### 2.2. Forming Process

#### 2.2.1. Melt Treatment

**Figure 1:**
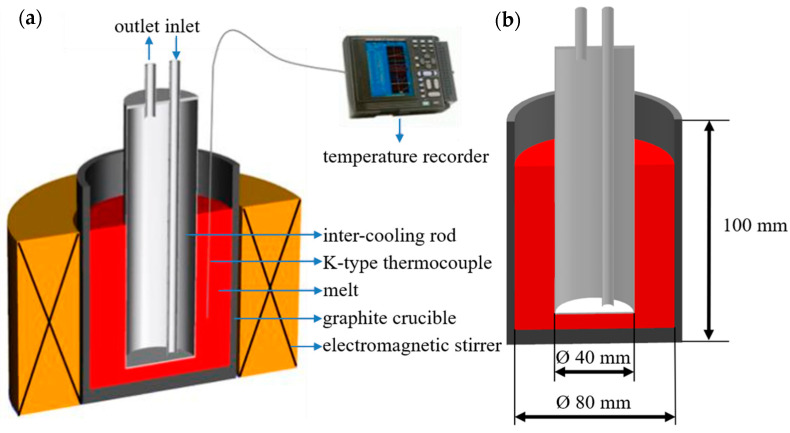
(**a**) Schematic view of the melt treatment apparatus by IC-AEMS, (**b**) the experimental dimensions of the graphite crucible and the inter-cooling rod [22].

In this paper, the IC-AEMS method and platform of Guan [22] are used to stir the self-configured 6.54Zn-2.40Cu-2.35Mg-0.10Zr(-Sc) melt. Figure 1a is the schematic diagram of the IC-AEMS melt processing device, and Figure 1b is the experimental size of the graphite crucible and the intermediate cooling rod.

**Figure 2:**
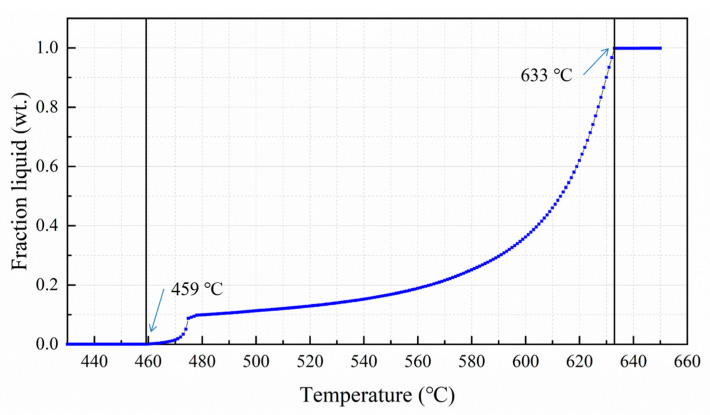
Liquid fraction of Al-6.54Zn-2.40Cu-2.35Mg-0.10Zr aluminum alloy with temperature calculated by JmatPro software.

The alloy raw materials were placed in a graphite crucible and heated to 750 °C in a box-type resistance furnace. After being completely melted, 0.5 wt.% hexachloroethane was used for degassing. After degassing, take out the crucible and remove the slag floating on the surface of the melt. The crucible was placed on the electromagnetic stirring platform as shown in Figure 1a for electromagnetic stirring. The operating parameters of the electromagnetic stirring platform were voltage 25 V, current 24 A, and frequency 15 Hz. According to the graph of the liquid fraction of Al-6.54Zn-2.40Cu-2.35Mg-0.10Zr aluminum alloy with temperature calculated by JmatPro software (Sente Software Ltd., Guildford, UK), as shown in Figure 2, considering the wide solid–liquid solidification interval and poor casting fluidity, the casting temperature should be above the liquidus line of 633 °C. While too high casting temperature is not conducive to grain refinement [7], the casting temperature was set to 660 °C—that is, the IC-AEMS melt treatment stopped when the temperature reached 660 °C, and then rheological die forging was performed.

#### 2.2.2. Rheological Die Forging

**Figure 3:**
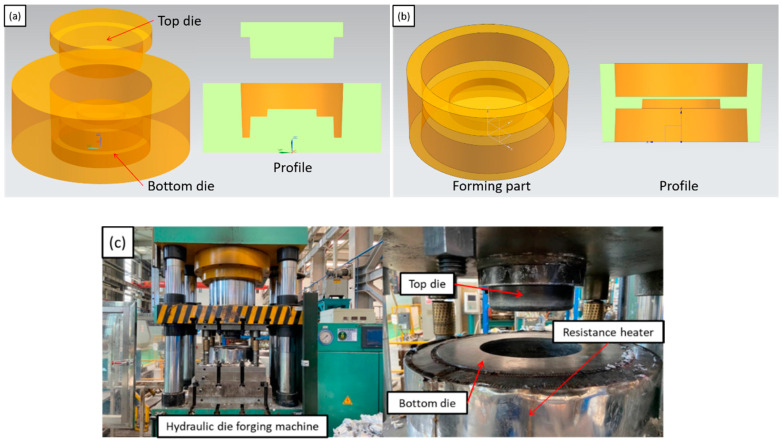
(**a**) Schematic diagram of the designed die; (**b**) schematic diagram of the forming part; (**c**) the actual die assembly and resistance heater.

Scale-reduced part model of brake hub for rheological die forging was designed and manufactured. The top and bottom dies are shown in Figure 3a, and the forming part is shown in Figure 3b. A resistance heater was used for die heating. The actual die assembly is shown in Figure 3c. The outer diameter of the part is 200 mm, the inner diameter is 170 mm, the height is 100 mm, and the lateral thickness is 15 mm. The draft angle is 2°. The dies were assembled on the LYF-400SA hydraulic press and preheated before rheological die forging. When the melt temperature reached the casting temperature of 660 °C, a quantitative amount of melt was poured into the cavity of the bottom die, and the hydraulic press was operated for rheological die forging. After formation, the part was taken out and cooled with air. Table 2 presents the rheological die forging process parameters.

### 2.3. Heat Treatment

Gao [23] and Fan [24] studied the changes in the microstructure of 7xxx aluminum alloy ingots during the homogenization process and found that the low melting point phase would melt during the heating process. According to the differential scanning calorimetry (DSC) analysis, two endothermic peaks appear when the 7xxx aluminum alloy is heated to 477 and 490 °C. Research on the heat treatment of 7xxx aluminum alloy mostly focuses on the homogenization of ingots and the solid solution and aging of forgings. As for the 7xxx directly casting near-net shape parts, the research on it is more scarce, and the heat treatment process can only refer to some previous research work. In industry, the homogenization temperature of 7xxx ingots generally does not exceed 470 °C to avoid over-burning, while 7xxx forgings have undergone the homogenization of ingots and plastic processing with large deformation before solid solution, and the solid solution temperature generally does not exceed 475 °C. Due to its near-net shape forming properties, the rheological die forging parts will no longer undergo plastic deformation. Therefore, for 7xxx rheological die forging parts, homogenization and solution treatment are regarded as the same process. Combined with the previous research, the T6 heat-treatment process of 6.54Zn-2.40Cu-2.35Mg-0.10Zr(-Sc) rheological die forging parts is determined as shown in Table 3. The solution treatment was in the box-type resistance furnace, the heating rate was 3 °C/min, and the quenching transfer time was less than 5 s. The aging treatment was in the drying box, and the heating rate was 3 °C/min. The aging treatment was 120 °C × 24 h to achieve T6 status, and the aging treatment was completed with furnace cooling.

### 2.4. Microstructure and Mechanical Properties Analysis

**Figure 4:**
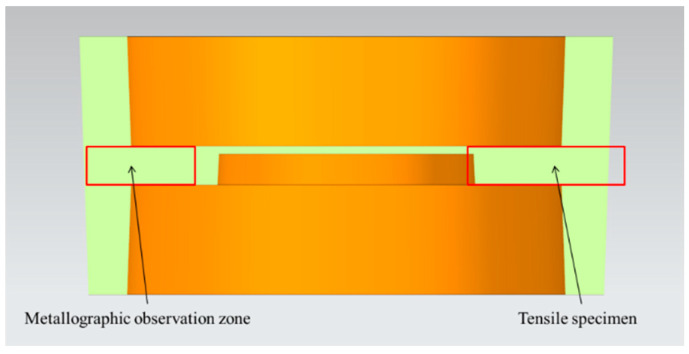
Metallographic observation surface and sampling position of tensile specimen.

**Figure 5:**
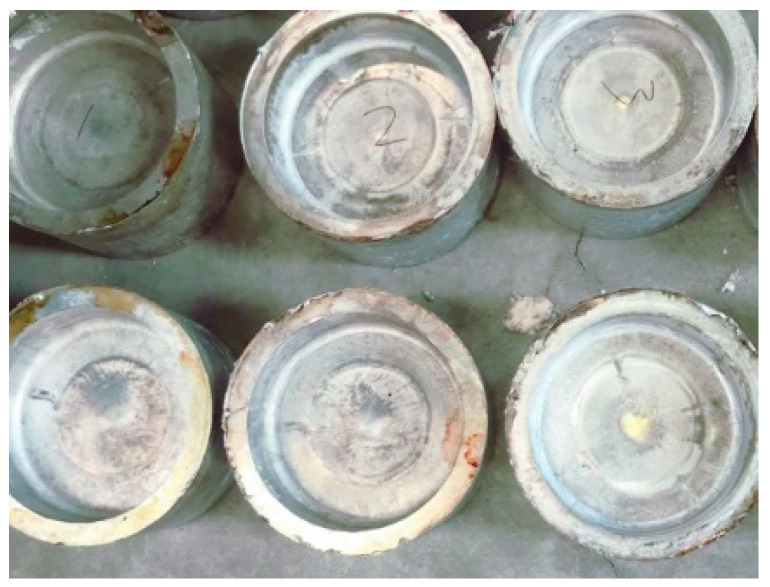
Scale-reduced parts of brake hub formed by rheological die forging.

**Figure 6:**
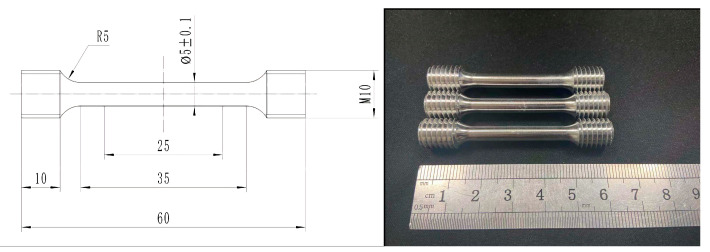
Schematic and physical diagram of dog-bone rod-shaped standard tensile specimen.

The sampling location and its function are shown in Figure 4. Figure 5 is a physical view of the scale-reduced parts of brake hub formed by rheological die forging, which were well formed and had no cracks on the surface. The tensile specimen was sampled from the transverse middle of the part and processed into a dog-bone rod-shaped standard pattern. The processing size is shown in Figure 6. The uniaxial tensile tests were performed at a constant tensile rate of 2 mm/min on a WDW-200 electronic universal testing machine. The ultimate tensile strength and elongation were directly read on the machine, and the stress value that produces 0.2% residual elongation was specified as the yield strength. In order to reduce the error, three tensile specimens were tested from each part, and the arithmetic mean value of mechanical property results was taken.

OM and SEM were used to observe the microstructure of the part. Before OM and SEM observation and EDS analysis, the observation surface was polished to achieve a mirror finish. In addition, the samples were anodic coated with a solution containing 5% HBF_4_ and 95% deionized water at 25 °C and 30 V DC voltage for 30 s, and the color metallography was observed under polarized light to distinguish the morphology and size of the grains. Zeiss Axiovert 200 MAT OM (Zeiss, Oberkochen, Germany) was used for optical metallographic observation, the higher magnification microstructure was observed by field emission SEM (JSM-7900F, JEOL Ltd., Tokyo, Japan), and the element composition and distribution of the observation area were analyzed by EDS.

## 3. Results

### 3.1. Evolution of Microstructure during Heat Treatment

**Figure 7:**
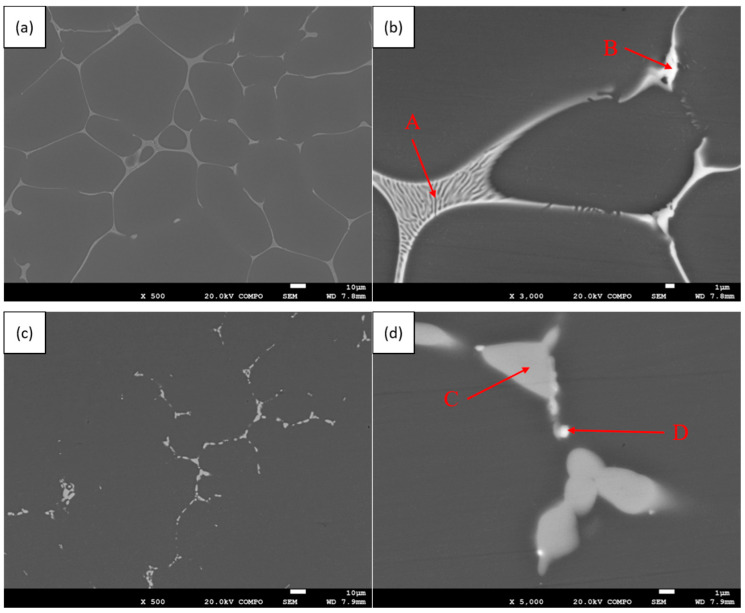
Scanning electron microscope photos of 1# in different heat treatment states, (**a**,**b**) as-cast, (**c**,**d**) TST-T6.

We observed the backscattered electron photos of 1# samples under different heat treatment conditions under a SEM, and analyzed the element distribution of each phase through EDS (The arrow points to an area with a diameter of about 1 μm EDS scan), as shown in Figure 7.

In the as-cast structure, the reticular eutectic phase with brighter contrast at the grain boundary is the AlZnMgCu quaternary phase, as shown at A in Figure 7b; the skeletal phase, which is brighter than the reticular eutectic phase, is the Cu-rich phase, as shown at B in Figure 7b. After TST-T6 heat treatment, the intergranular phase decreases and transforms from a gray network shape to a gray chain bead shape, which can be determined as S phase after EDS analysis, as shown at C in Figure 7d; and there are bright spots around the S phase, as shown at D in Figure 7d, which is the Cu-rich phase. The chemical components and possible phases indicated by the arrows in Figure 7 are shown in Table 4.

The intergranular phase in the as-cast microstructure is mainly the AlZnMgCu quaternary phase, which is composed of two infinitely miscible T phases (Al_6_CuMg_4_ + Al_2_Mg_3_Zn_3_). Due to component segregation, Cu elements are enriched in some locations, and Al_2_Cu phase is first precipitated during the solidification process, which is accompanied by T phase precipitated later. After TST-T6 heat treatment, due to the lower melting point of the T phase, the higher solubility of Zn re-dissolves into the aluminum matrix, and the remaining element diffusion capacity is limited, forming the higher melting point S phase, while the higher melting point Al_2_Cu has almost no change, and is associated with S in the grain boundary. Due to the limited resolution of SEM, the Zr-rich phase is not observed, and can only be detected indirectly by EDS. Interestingly, the Zr-rich phase is more likely to precipitate in or with the Cu-rich phase.

### 3.2. OM Observation

**Figure 8:**
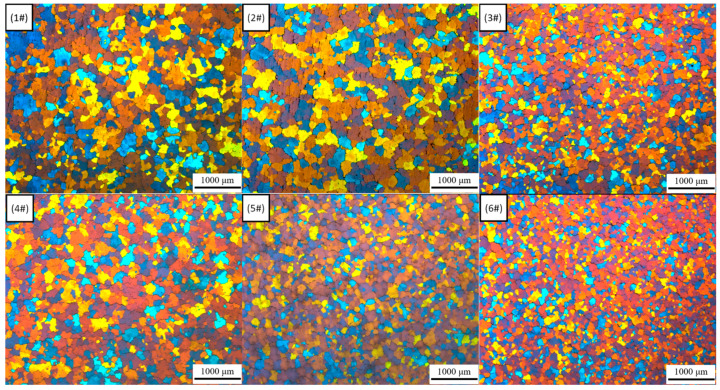
Color metallographic photos 1–6#.

Figure 8 presents color metallographic photos of the microstructure of 1–6# in TST-T6 status. Observed from the color metallographic photos, it is obvious that adding Sc element and applying IC-AEMS to the melt can refine the grains.

**Figure 9:**
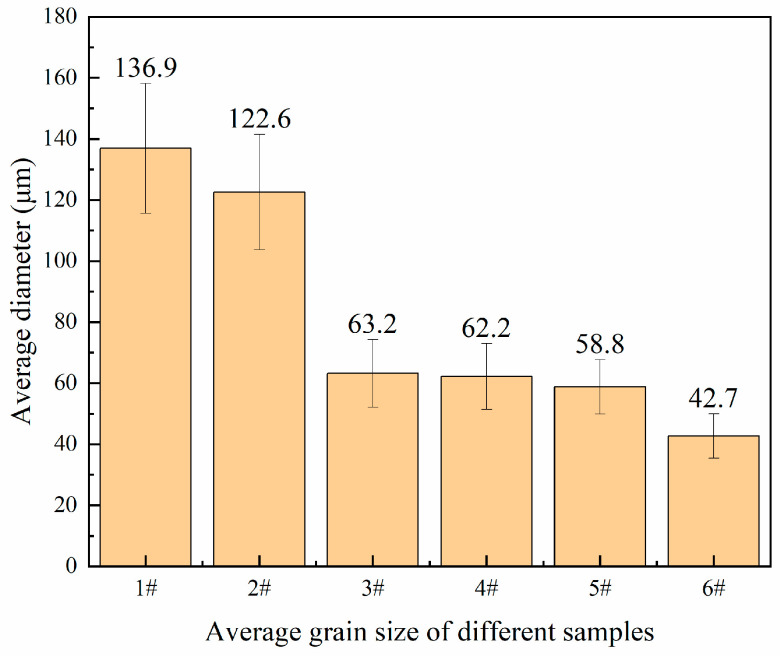
Average grain size of each sample.

We counted the average grain size of each group of samples with the cross-line method. The calculation formula is $\bar{d}=L/N$, where $\bar{d}$ is the average grain size, and L is the total length of the line in the field of view (actual length/magnification), N is the total number of grains crossed by the line. The statistical results of the grain size of all samples are shown in Figure 9.

### 3.3. Mechanical Performance

**Figure 10:**
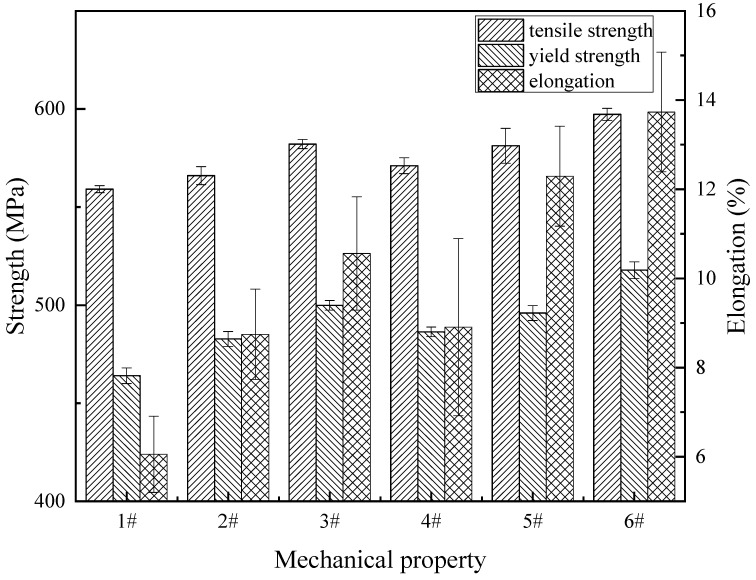
Static tensile mechanical properties of all samples.

We took the samples as shown in Figure 4 and processed them into the tensile specimens shown in Figure 6. The ultimate tensile strength, yield strength and elongation were obtained through static tensile tests. The results are shown in Table 5. The standard deviation is expressed in the form of error bars in the subsequent analysis, which is shown in the form of a histogram, as shown in Figure 10.

## 4. Discussion

### 4.1. Effect of Sc on the Microstructure and Mechanical Properties

Comparing the grain size and mechanical properties of samples 1#, 2#, and 3#, with the increase in Sc content, the average grain size decreased from 136.9 μm to 63.2 μm, while the ultimate tensile strength, yield strength and elongation were all improved. Taking the average result of these mechanical properties and calculating the improvement rate, the results of calculation are shown in Table 6.

**Figure 11:**
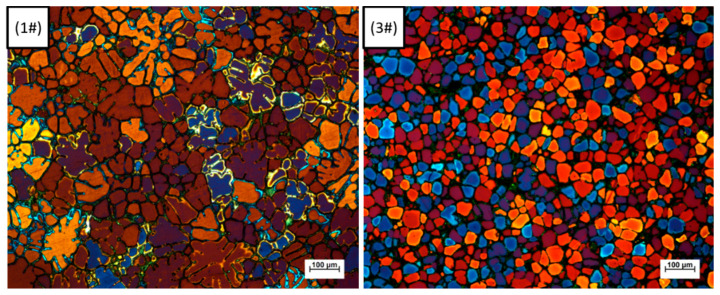
Color metallographic photos of the microstructure of 1# and 3# in as-cast status.

Compared with the sample without adding Sc, the addition of 0.1 wt.% Sc increased the ultimate tensile strength, yield strength and elongation by 1.23%, 4.05% and 42.62%, respectively, while the addition of 0.15 wt.% Sc increased the ultimate tensile strength and yield. The strength and elongation were increased by 4.11%, 7.74% and 73.77%, respectively. Referring to the results in Figure 8, the addition of Sc can significantly reduce the grain size, and the grain refinement can significantly increase the elongation of the material. According to the Hall–Petch formula:

(1) $${\sigma =\sigma }_{0}{+k}_{y}d^{-0.5},$$

where σ_0_ is the intrinsic stress, d is the average grain size, and k_y_ is the Hall–Petch coefficient. For Al-Zn-Mg-Cu alloy, k_y_ is approximately 0.22 MPa/m^0.5^ [25]. Given the value of d from Figure 9, the increase in yield strength from 1# to 3# due to grain refinement is estimated to be 8.87 MPa. A recent study showed that for Al-Zn-Mg-Cu alloy, both grain boundary strengthening and precipitation strengthening are dominant mechanisms after T6 heat treatment [25]. The introduction of Sc will generate Al_3_(Sc, Zr) particles dispersed in the matrix [26], resulting in precipitation strengthening. Figure 11 presents color metallographic photos of the microstructure of 1# and 3# in as-cast status.

According to Figure 11, after adding 0.15 wt.% Sc, the morphology of the as-cast crystal grains changed from dendritic to equiaxed cellular crystals. According to research [26], the addition of Sc to the Zr-containing high-strength aluminum alloy will significantly reduce the grain size. When the Sc content exceeds the critical value, the primary Al_3_(Sc, Zr) phase will be formed in the structure, which acts as an effective nucleating agent, but the coarse primary Al_3_(Sc, Zr) phase is not conducive to the tensile properties of the material. Another study [27] concluded that the strength of aluminum alloy increases with the increase in Sc content. When the Sc content reaches 0.3 wt.%, the aluminum alloy has the highest strength. However, due to the small particle spacing of Al_3_Sc particles, it has a strong inhibitory effect on the dislocation movement, resulting in a decrease in elongation. Research [28] showed that when adding Sc to the Zr-containing alloy to refine the grain, the Sc content should be reduced accordingly. Zr can enter the Al_3_Sc lattice and partially replace Sc atoms to form replacement solid solution Al_3_(Sc_1−x_, Zr_x_) particles. Its thermal stability and tendency to aggregate and grow at high temperatures are related to the content of Zr atoms. Research [29] shows that the higher the value of x, the smaller the tendency of Al_3_(Sc_1−x_, Zr_x_) particles to aggregate and grow, and Zr atoms can replace up to 50 wt.% of Sc atoms.

In this experiment, the mass proportion of Sc and Zr elements does not exceed 0.25 wt.%, which is not enough to produce coarse Al_3_(Sc, Zr) primary phases while refine and equiaxialize the grains, which enables the material to produce grain boundary strengthening and precipitation strengthening at the same time. Larger grain boundary density and fine precipitates make it easier for the material to hinder the movement of dislocations between and inside the grains, which improves the strength of the material. Under the same amount of deformation, the increase in the number of equiaxed grains per unit volume results in a more dispersed and uniform deformation, fewer dislocations accumulate inside a single grain, and there is less chance of cracking due to stress concentration, which means the material can withstand a greater amount of deformation before cracking—that is, the increase in elongation.

### 4.2. Effect of Electromagnetic Melt Treatment on the Microstructure and Mechanical Properties

Comparing the grain size and mechanical properties of samples 4#, 5#, and 6# with those of 1#, 2#, and 3#, the minimum average grain size has been further reduced, from 63.2 μm in 3# to 42.7 μm in 6#. According to Figure 9, After the application of IC-AEMS, the ultimate tensile strength, yield strength and elongation rate are improved compared with samples without electromagnetic stirring, and the results of the improvement rate are shown in Table 7.

**Figure 12:**
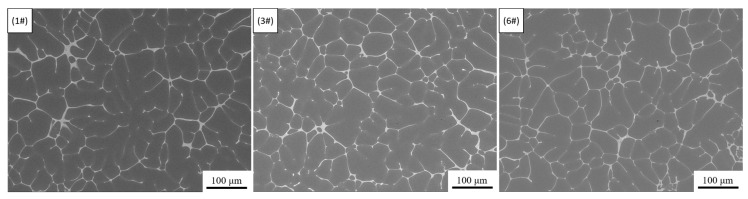
SEM backscattered photographs of 1#, 3# and 6# in as-cast status.

Compared with the sample without electromagnetic stirring, the application of IC-AEMS increased the ultimate tensile strength, yield strength and elongation by an average of 2.48%, 3.71%, and 38.84%, respectively. This means that IC-AEMS can further enhance the strengthening effect of Sc on the alloy. Figure 12 is a SEM backscattered photograph of 1#, 3# and 6# in as-cast status.

According to Figure 12, the intergranular T phase structure is uniformly distributed, and no Sc and Zr-containing phases are observed in and between the grains. We used graphic processing software to make statistics on the content of the intergranular phase, which in 1#, 3# and 6# is 7.71%, 7.24% and 6.38%, respectively. The results show that the content of the intergranular phase is slightly reduced after adding 0.15 wt.% Sc, while the content of intergranular phase is further reduced after applying IC-AEMS. The smaller the content of the second phase, the smaller the degree of micro-segregation—that is, electromagnetic stirring can further reduce the micro-segregation of the as-cast structure, which is beneficial to the subsequent heat-treatment and can make the alloying elements more fully dissolved into the aluminum matrix.

Research [30] showed that when the aluminum alloy melt is subjected to forced shearing by electromagnetic stirring, the temperature field and atomic cluster distribution of the alloy melt are relatively uniform, and the nucleation occurs simultaneously in the entire melt, reaching an “explosive type” nucleation, which changes the growth mode of crystal nuclei from competing with each other to growing close to the same time, resulting in a uniform and fine solidified structure. Due to the effect of the electromagnetic field, the refined grains increase the total number of grains and the grain boundary area, which promotes the transformation of the morphology of the grains from coarse dendrites to equiaxed cellular grains. This reduces the network structure formed by the development of dendrites, and avoids the formation of a coarse eutectic phase during the solidification process [31], which reduce the micro-segregation between grains. Research [20] shows that in the process of melt cooling, the crucible wall with a lower temperature provides undercooling for the nucleation of Al_3_(Sc, Zr) particles, and electromagnetic stirring makes the temperature field composition field more uniform, which can increase Al_3_(Sc, Zr) particle stability. Research [32] shows that IC-AEMS has both the advantages of rapid cooling and uniform temperature field and composition field, and the role of the inter-cooling rod is similar to the crucible wall, which can provide the melt with more undercooling required for Al_3_Zr nucleation, and IC-AEMS can inhibit the growth rate of Al_3_Zr particles. Therefore, for the Sc-containing 7050 alloy in this paper, IC-AEMS can form more Al_3_(Sc, Zr) nucleating particles, and further refine the grains.

### 4.3. Fracture Analysis

**Figure 13:**
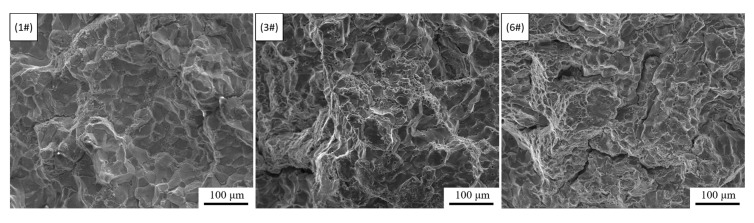
SEM photos of tensile fractures of 1#, 3# and 6#. (1#: no treatment; 3#: no electromagnetic stirring, adding 0.15 wt.% Sc; 6#: applying IC-AEMS, adding 0.15 wt.% Sc).

The tensile fractures of 1#, 3#, and 6# were observed by SEM, and the results are shown in Figure 13.

A lot of rock candy-shaped particles can be observed in the tensile fracture of sample #1, showing the characteristics of intergranular fracture; and a small part of planes and tearing edges shows quasi-cleavage characteristics. These characteristics indicate that the fracture mode of the sample is a mixed brittle fracture, including mainly intergranular fracture and some quasi-cleavage transgranular brittle fracture. The 1# sample has not undergone any treatment, so the crystal grains are relatively coarse, the distribution of the second phase between the crystals is uneven, and the size is large, which is distributed in a network after heat treatment. Under the action of external load, the intergranular network brittle phase is directly subjected to the load and is easily broken to form cracks and cause the cracks to propagate along the grain boundaries, forming brittle fractures along the grains.

A large number of quasi-cleavage fracture characteristics of the tearing edge, pits and facets surrounded by it, and small dimples appeared in the tensile fracture of the 3# sample. No rock candy-shaped particles were found. These characteristics indicate that the fracture mode of the sample is a transgranular ductile fracture dominated by quasi-cleavage fracture. After adding 0.15 wt.% Sc element, the grains are refined and equiaxed, the intergranular phases are reduced, and the grain boundaries increase, making it more difficult for cracks to propagate along the grain boundaries. Under the action of external load, the dislocation pinned and plugged at the hard phases between and in the grains during the migration process, and finally produced microcracks. However, due to the refinement of the grains, the dislocations in each grain are less dense than the coarse grains under the same amount of deformation, so the sample can withstand greater deformation before the microcracks propagate to be able to break, which increases the plasticity of the material.

In the tensile fracture of the 6# sample, the quasi-cleavage fracture characteristic can also be observed, but the dimples are more distributed and larger in size, showing more obvious characteristics of transgranular ductile fracture. Through the analysis of 4.2, IC-AEMS can further refine the crystal grains and reduce the micro-segregation between the crystals. Therefore, compared with the 3# sample, the 6# sample shows a more obvious fine-grain strengthening effect.

## 5. Conclusions

In this work, a scale-reduced part of a brake hub for rheological die forging was designed. By adding Sc element to the melt and applying electromagnetic stirring, the scale-reduced parts of a brake hub with different microstructure characteristics were successfully prepared by rheological die forging process. The microstructure evolution and mechanical properties of Al-6.54Zn-2.40Cu-2.35Mg-0.10Zr(-Sc) rheological die forging parts were studied through different material characterization methods. The conclusions drawn from the experimental observation are as follows:

1. Al-6.54Zn-2.40Cu-2.35Mg-0.10Zr aluminum alloy as-cast structure mainly contains intergranular T phase, after the TST-T6 heat-treatment, part of the T phase transforms into the S phase and remains between grains;
2. The addition of 0.15 wt.% Sc to the Al-6.54Zn-2.40Cu-2.35Mg-0.10Zr aluminum alloy can significantly reduce the grain size and transform the grain morphology from dendrites to equiaxed cellular grains without producing an Al_3_(Sc, Zr) primary phase, improving the strength and plasticity of the material at the same time;
3. IC-AEMS melt-treatment can further enhance the Sc refinement effect, and can reduce the micro-segregation of solute elements between the grains, which reduces the formation of intergranular eutectic phases;
4. Through rheological die forging technology, the Al-6.54Zn-2.40Cu-2.35Mg-0.10Zr(-Sc) aluminum alloy scale-reduced parts of brake hub was successfully prepared, the structure is compact and defect-free, and well-formed. By adding 0.15 wt.% Sc and IC-AEMS melt processing, the ultimate tensile strength of the parts is 597.2 ± 3.1 MPa, the yield strength is 517.8 ± 4.3 MPa, and the elongation is 13.7 ± 1.3% after TST-T6 heat treatment, reaching the normal performance level of 7xxx aluminum alloy forgings, realizing the near-net shape forming of high-strength aluminum alloy.

These results help to control and improve the microstructure of aluminum alloy castings, and provide ideas and methods for the near-net shape forming of 7xxx series high-strength aluminum alloy parts. This work only studied the influence of Sc element and electromagnetic stirring technology on the microstructure and mechanical properties, and did not investigate the influence of the forging process parameters on the microstructure and mechanical properties. In the future work, the influence of different forging process parameters on the microstructure and mechanical properties should be investigated, so as to obtain the process parameters that make the parts get the best performance, and guide the mass production of subsequent parts. The multi-stage high-temperature solution heat treatment process should be further explored to minimize the amount of residual second phases and to maximize the material’s better mechanical properties on the premise of avoiding microstructure over-burning.

## Figures and Tables

**Table 1:** Composition of high alloyed Al-Zn-Mg-Cu aluminum alloy.

Elements	Zn	Mg	Cu	Zr	Fe	Si	Al
wt.%	6.54 ± 0.05	2.35 ± 0.02	2.40 ± 0.04	0.103 ± 0.001	<0.001	<0.003	Bal.

**Table 2:** Rheological die forging process parameters.

No.	Die Temperature/°C	Casting Temperature/°C	Compress Time/s	Working Speed /mm·s^−1^	Specific Pressure /MPa	Melt Treatment	wt.% (Sc)
1#	200	660	5	20	100	None	0
2#	200	660	5	20	100	None	0.1
3#	200	660	5	20	100	None	0.15
4#	200	660	5	20	100	IC-AEMS	0
5#	200	660	5	20	100	IC-AEMS	0.1
6#	200	660	5	20	100	IC-AEMS	0.15

**Table 3:** Heat treatment of 7050 rheological die forging parts.

Solution Process	Solution Temperature and Time
Two-stage solution treatment (TST)	460 °C × 3 h + 475 °C × 3 h

**Table 4:** EDS analysis of each phase indicated by the arrow in Figure 7. (at.%).

Destination	Al	Zn	Mg	Cu	Zr	Fe	Si	Possible Phase
A	70.35	8.14	14.4	6.95	0.15	-	-	T (Al_6_CuMg_4_ + Al_2_Mg_3_Zn_3_)
B	68.73	4.63	8.42	16.70	0.97	-	0.54	T (Al_6_CuMg_4_ + Al_2_Mg_3_Zn_3_) + θ (Al_2_Cu)
C	65.51	1.46	18.23	14.66	0.13	-	-	S (Al_2_CuMg)
D	80.11	1.87	5.81	10.22	1.35	0.25	0.39	S (Al_2_CuMg) + θ (Al_2_Cu) + Al_3_Zr

**Table 5:** Average results of static tensile test of all samples.

No.	Ultimate Tensile Strength/MPa	Yield Strength/MPa	Elongation/%
1#	559.1 ± 1.8	464.0 ± 4.0	6.1 ± 0.9
2#	566.0 ± 4.6	482.8 ± 3.8	8.7 ± 1.0
3#	582.1 ± 2.3	499.9 ± 2.5	10.6± 1.3
4#	571.1 ± 4.0	486.4 ± 2.5	8.9 ± 2.0
5#	581.2 ± 8.9	496.0 ± 3.8	12.3 ± 1.1
6#	597.2 ± 3.1	517.8 ± 4.3	13.7 ± 1.3

**Table 6:** The improvement rate of mechanical properties by adding Sc element. (%).

Sc Content/wt.%	Ultimate Tensile Strength	Yield Strength	Elongation
0.1	1.23	4.05	42.62
0.15	4.11	7.74	73.77

**Table 7:** The improvement rate of mechanical properties by applying IC-AEMS. (%).

Mechanical Property	0Sc	0.1 wt.% Sc	0.15 wt.% Sc	Average
Ultimate tensile strength	2.15	2.69	2.59	2.48 ± 0.29
Yield strength	4.83	2.73	3.58	3.71 ± 1.06
Elongation	45.90	41.38	29.25	38.84 ± 8.61
